# Homozygous truncating mutation in *NRAP* gene identified by whole exome sequencing in a patient with dilated cardiomyopathy

**DOI:** 10.1038/s41598-017-03189-8

**Published:** 2017-06-13

**Authors:** Grażyna T. Truszkowska, Zofia T. Bilińska, Angelika Muchowicz, Agnieszka Pollak, Anna Biernacka, Katarzyna Kozar-Kamińska, Piotr Stawiński, Piotr Gasperowicz, Joanna Kosińska, Tomasz Zieliński, Rafał Płoski

**Affiliations:** 1 Molecular Biology Laboratory, Department of Medical Biology, Institute of Cardiology, Warsaw, Poland; 2 Unit for Screening Studies in Inherited Cardiovascular Diseases, Institute of Cardiology, Warsaw, Poland; 3 Department of Immunology, Medical University of Warsaw, Warsaw, Poland; 4 Department of Genetics, Institute of Physiology and Pathology of Hearing, Warsaw, Poland; 5 Department of Medical Genetics, Medical University of Warsaw, Warsaw, Poland; 6 Postgraduate School of Molecular Medicine, Warsaw, Poland; 7 Immunology Laboratory, Department of Medical Biology, Institute of Cardiology, Warsaw, Poland; 8 Department of Heart Failure and Transplantology, Institute of Cardiology, Warsaw, Poland

## Abstract

The genetic background of dilated cardiomyopathy is highly heterogeneous, with close to 100 known genes and a number of candidates described to date. Nebulin-related-anchoring protein (NRAP) is an actin-binding cytoskeletal protein expressed predominantly in striated and cardiac muscles, and is involved in myofibrillar assembly in the foetal heart and in force transmission in the adult heart. The homozygous *NRAP* truncating variant (rs201084642), which is predicted to introduce premature stop codon into all NRAP isoforms, was revealed in the dilated cardiomyopathy patient using whole exome sequencing. The same genotype was detected in the asymptomatic proband’s brother. The expression of the NRAP protein was undetectable in the patient’s heart muscle by the Western blot. Genotyping for rs201084642 in the ethnically matched cohort of 231 dilated cardiomyopathy patients did not reveal any additional subjects with this variant. Our findings suggest that the biallelic loss-of-function mutation in *NRAP* could constitute a relatively rare, low-penetrance genetic risk factor for dilated cardiomyopathy.

## Introduction

Nebulin-related anchoring protein (NRAP), expressed in the heart and in striated muscles, is the second largest member of the actin-binding cytoskeletal proteins from nebulin family (193–196 kDa in humans)^1^. NRAP is expressed in myofibril precursors during myofibrillogenesis^2, 3^. In adult mice and humans NRAP expression is limited to: (i) terminal bundles of actin filaments at the myotendinous junctions of skeletal muscles, and (ii) the intercalated discs of the heart muscles and is not observed in mature sarcomeres^1, 4–6^. NRAP is composed of a LIM domain followed by 46 nebulin repeats (11 simple repeats and 5 super repeats each consisting of 7 repeats)^1, 5^. The NRAP N-terminal LIM domain was shown to interact with α-actinin and talin^6, 7^ while the domain with single repeats interacts with actin^7^, α-actinin and the Kelch-like family member 41 (KLHL41, also known as Krp1)^8^, as well as cysteine and glycine-rich protein 3 (CSRP3), also known as muscle LIM protein (MLP)^9^. C-terminal super repeats of NRAP interact with filamin C^8^ and vinculin^7^. During cardiomyocyte development in the foetal heart, NRAP is involved in myofibrillar assembly^3^ and links terminal actin filaments to membrane complexes in the adult heart (thus potentially playing a role in force transmission from the sarcomere to the extracellular matrix)^1, 7^. In mice, heart-specific 2.5-fold overexpression of NRAP leads to right ventricular cardiomyopathy^10^. The expression of NRAP is upregulated in mice models of dilated cardiomyopathy (DCM), such as CSRP3/MLP knockout mice and tropomodulin overexpressing transgenic mice^9^. The upregulation of NRAP is also observed in human DCM patients^11^.

Despite evidence that NRAP plays a role in both in the developing heart and the adult heart, no variants in this gene have been so far conclusively associated with cardiac disease.

## Results

### Clinical findings

The proband was a 26-year-old patient, healthy until December 2014, who developed rapidly progressive symptoms of biventricular heart failure after a prolonged viral-like illness. On admission to our institute, he was in a low cardiac output state requiring intravenous dobutamine support. He had on a 12-lead standard electrocardiogram, a sinus rhythm of 115 per minute, normal QRS axis of +2°, left atrial enlargement, PR 150 ms and QRS 88 ms, and a borderline prolonged QT interval was found with QTc 475 ms. There was poor r wave progression in leads V1-V3. The CK serum level was low normal at 68 U/l (N: 39–308). His NT-proBNP was 5154 pg/ml, and hs troponin T was 15.04 ng/ml; he had normal thyroid function tests and a calcium/phosphorus level, and a low normal iron level in serum. On the two-dimensional Doppler echocardiogram, an enlarged left ventricle of 71 mm with global diffuse hypokinesis was found (LVEF of 15%) along with an enlarged left atrium with severe mitral insufficiency. The cardiac magnetic resonance study global found global diffuse hypokinesis of both ventricles (LVEF and RVEF of 19%), significantly increased left ventricular volume LVEDVI 223 ml/m^3^ (N: 68–103 ml/m^3^), and mildly increased RV volume RVEDVI 129 ml/m^3^ (N: 68–114 ml/m^3^). Maximal heart muscle thickness was 10 mm, and LV mass was normal at 78 g/m^2^ (N: 59–93 g/m^2^). Both atria were enlarged, the left atrium significantly at 40.8 cm^2^ and the right atrium at 24 cm^2^. There was significant mitral insufficiency with the restricted position of mitral leaflets and no coaptation; the mitral regurgitation volume was 48 ml and regurgitation fraction 56%. In addition, one focus of late gadolinium enhancement was identified in the low junction of the right and left ventricles. With progressive symptoms and increasing dependence on catecholamines, the patient was referred for heart transplantation and was successfully transplanted four months after the onset of symptoms. While on the active list for HTX the patient experienced one episode of sustained ventricular tachycardia treated with electric cardioversion, which was followed by ICD-VR implantation in secondary prevention.

In the family, there is no history of heart failure, sudden cardiac death, or cardiac arrhythmia among first-degree and second-degree relatives. The proband’s father (I:1, Fig. 1A), examined at 67 years of age, was treated for hypertension and articular psoriasis and had a normal 12-lead electrocardiogram; however, he also had a high normal QTc of 464 ms and normal two-dimensional Doppler echocardiogram. At the age of 53 years the proband’s father was operated on because of a subarachnoid haemorrhage related to an aneurysm in the medial cerebral artery, without complications. The proband’s mother (I:2), aged 60 years was asymptomatic, obese (BMI 33 kg/m^2^) and with normal non-invasive cardiological tests. In addition, we examined the two available asymptomatic siblings, the 36- year-old brother (II:3) and the 31-year-old sister (II:5). Both of them had normal echocardiographic examinations. In the proband’s sister, short PR of 99 ms; and QTc of 442 ms were noted. In the remaining relatives, both PR 148–158 ms and QTc 410–420 ms were normal.

**Figure 1:**
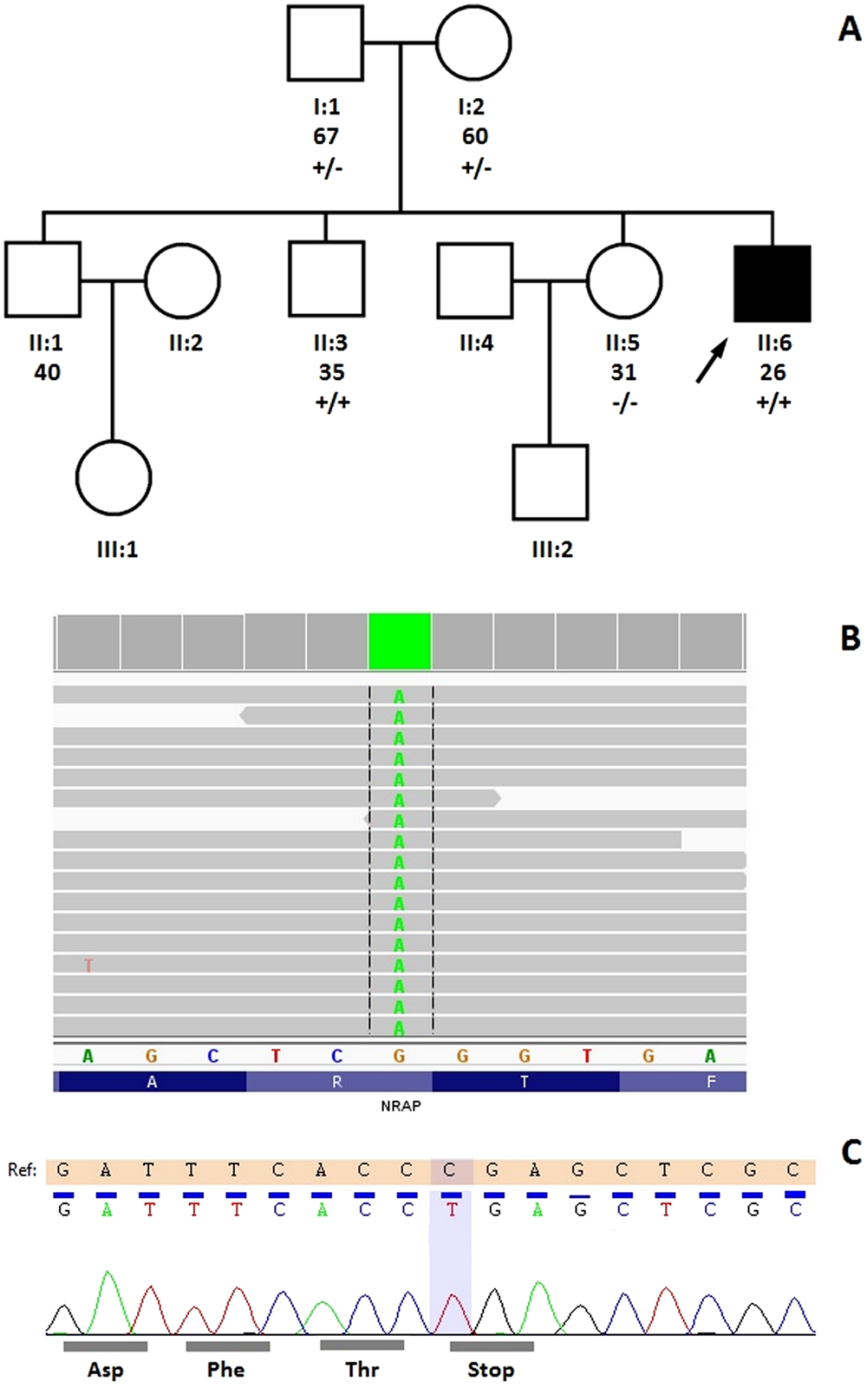
The *NRAP* NM_198060.3:c.4504C > T:p.Arg1502* variant in the proband. Pedigree of proband family (**A**) and IGV view of the *NRAP* NM_198060.3:c.4504C > T:p.Arg1502* variant found by whole exome sequencing (**B**) and chromatogram from direct sequencing by the Sanger method (**C**). Pedigree: squares represent males and circles represent females. An arrowhead denotes the proband. Solid black symbols denote dilated cardiomyopathy and open symbols unaffected individuals. The presence or absence of the *NRAP* variant is indicated by a +/− symbol.

### Molecular findings

The sequencing run for the patient sample achieved 127 925 372 reads with a mean coverage of 129.9. Above 94% and 97% of the target region was covered a minimum 20 and 10 times respectively. Initial search for variants located in the coding or splicing regions of the established cardiomyopathy/myopathy genes with a frequency no greater than 0.01 in all four databases (1000Genomes, ESP6500, ExAC, POL400-database of >400 exomes from Polish individuals) did not reveal variants of note. The second search was not limited to known genes and included variants located in the coding or splicing regions with a frequency no greater than 0.01 in all four databases (1000Genomes, ESP6500, ExAC, POL400). By this filtering, we obtained a list of 420 variants, of which 17 were loss-off-function (LoF) including two variants in *NRAP* and basic salivary proline-rich protein 4 genes (*PRB4*) predicted to lead to nonsense-mediated decay of erroneous transcripts according to the Nagy and Maquat rule^12^. The homozygous *NRAP* LoF variant (rs201084642, Fig. 1B,C) was chosen as a candidate given its function and expression mainly in the heart and skeletal muscles. rs201084642 is predicted to affect all *NRAP* isoforms NM_006175.4:c.4399C > T:(p.Arg1467*), NM_198060.3:c.4504C > T:(p.Arg1502*), NM_001261463.1:c.4504C > T:(p.Arg1502*) leading to a premature stop codon located in the C-terminal part of the protein – in the super repeat 5 – and removing a significant (13%) portion of the protein. The rs201084642 variant has a frequency of 0.0002636 (32/121382 alleles) in the ExAC database and 0.0037736 (4/1060) in our in-house database. In this in-house database, the heterozygous *NRAP* rs201084642 variant was found in an additional four individuals (three probands with non-cardiac phenotype and one with arrhythmogenic right ventricular cardiomyopathy (ARVC) carrying pathogenic/likely pathogenic *PKP2* NM_004572.3:c.2489 + 1 G > A variant (rs111517471)).

**Figure 2:**
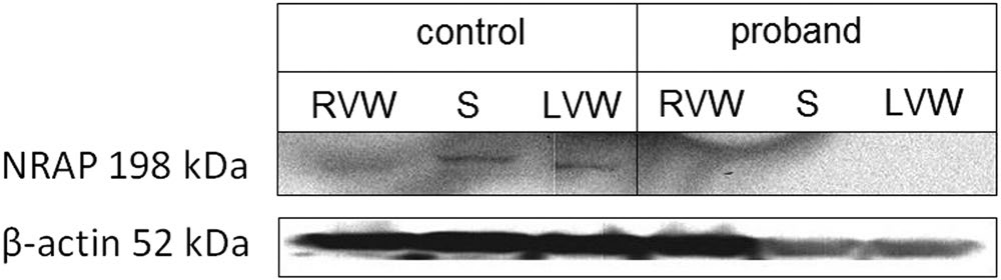
Western blot of NRAP protein from heart samples of proband and control. RVW - right ventricular wall, S - septum, LVW - left ventricular wall. Control sample was taken from heart transplantation recipient without truncating variants in *NRAP* gene.

SNP genotyping using TaqMan probes for rs201084642 in a cohort of 231 unrelated DCM patients of Polish origin did not reveal any additional homozygotes or heterozygotes for this variant. Direct Sanger sequencing for the *NRAP* NM_198060.3:c.4504C > T:p.Arg1502* variant in the proband’s family revealed heterozygosity for this variant in the proband’s non-consanguineous parents (I:1, I:2), wild type phenotype in his sister (II:5), and homozygosity for this variant in his older asymptomatic brother (II:3) (Fig. 1A). The NRAP protein in the proband’s heart samples from right and left ventricular wall and interventricular septum was undetectable by the Western blot, although it was clearly seen in a control heart (Fig. 2).

## Discussion

Our study is the first description of a DCM patient with homozygous truncating mutation in the *NRAP* gene. The *NRAP* NM_198060.3:c.4504C > T:p.Arg1502* (rs201084642) variant is present in the ExAC database with low frequency (AF-0.0002636); however, no homozygous LoF variants were reported in the ExAC or in the literature. The *NRAP* mutation is predicted to remove a significant part (13%) of the full-length protein and affect all NRAP isoforms. In agreement with this, we did not observe any NRAP protein expression by the Western blot in the patient’s heart. Taking into account that NRAP functions in the embryonic and adult hearts and that rs201084642 is rare, homozygous and leads to loss of function we postulate that a biallelic *NRAP* mutation could have contributed to cardiac dysfunction in our patient, perhaps in conjunction with viral illness and/or other genetic/non-genetic factors. Numerous studies indicate the influence of inflammatory reaction and genetic polymorphisms in heart failure. The utility of various biochemical and genetic biomarkers is tested to determine disease aetiology, predict disease progression and assess optimal medication^13–16^.

Homozygosity for rs201084642 was also found in the proband’s asymptomatic brother (36 years old), which indicates that LoF of NRAP in humans can be tolerated, i.e. the rs201084642 variant has limited penetrance and/or requires other factors to cause disease. The notion that NRAP deficiency is not pathogenic by itself is consistent with the recent report by D’Avila *et al*.^17^, who described an Italian female patient with myofibrillar myopathy caused by the *BAG3*:c.626C > T (p.P209L) mutation accompanied by three *NRAP* nonsynonymous variants (rs200747403 c.3674G > A:p.A1225V, rs2270182 c.1556T > A:p.N519I, rs2275799 c.844C > T:p.A282T) and the *FHL1* variant (rs151315725 c.823G > A:p.D275N). The muscle biopsy specimen from the D’Avila patient showed reduced NRAP mRNA levels and Western blot and immunohistochemistry showed a lack of the NRAP protein.

The *NRAP* rs201084642 variant is apparently not a frequent cause of cardiomyopathy in our population as we failed to find any additional positive subjects after screening a cohort of 231 DCM patients. This observation is consistent with the study by Mohiddin *et al*.^5^, who screened 50 patients with DCM or hypertrophic cardiomyopathy (HCM) which progressed to DCM for mutations in the *NRAP* gene and two members of a family with DCM linked to locus 10q24–26, and did not find any variants that could be classified as pathogenic.

In conclusion, we report the first patient with DCM with a homozygous LoF variant in the *NRAP* gene, as well as his healthy brother with the same genotype. Our findings suggest that biallelic LoF mutations in *NRAP* could constitute a low penetrance genetic risk factor for DCM.

## Methods

### Informed consent

The study was conducted according to the Declaration of Helsinki Principles. Written informed consent was obtained from the participants, and the study protocol (registration number 1457) was approved by the Bioethics Committee of the Institute of Cardiology, Warsaw, Poland.

### Genetic testing

DNA was extracted from the peripheral blood by standard salting out method or phenol extraction and from buccal swabs using Maxwell® 16 instrument with DNA IQ™ Casework Pro Kit (Promega). Library preparation was performed using SureSelect QXT V5 All Exome kit (Agilent Technologies, Cedar Creek, TX, USA) according to manufacturer instruction and sequenced on Illumina HiSeq1500 as described previously^18^.

### Whole Exome Sequencing (WES) data analysis

Variants from WES were filtered such that only coding/splicing variants with frequency no gather than 0.01 in: 1000 Genomes, Exome Variant Server, ExAC and POL400 (in-house database of >400 ethnically matched exomes) databases. Choice of variants for further analysis were limited to genes mainly expressed in skeletal and cardiac muscle and supported by *in silico* analysis by different bioinformatics tools: (PolyPhen2, SIFT, Combined Annotation Dependent Depletion (CADD), Mutation Assessor, the likelihood ratio test (LRT), FATHMM, Mutation Tester.

### Sanger sequencing

The *NRAP* NM_198060.3: c.4504C > T:p.Arg1502* (rs201084642) variant was followed-up in a proband and his relatives with Sanger sequencing using using a 3500xL Genetic Analyzer (Life Technologies, Carlsbad, CA, USA) and BigDye Terminator v3.1 Cycle Sequencing Kit (Life Technologies) according to the manufacturer’s instructions (primers forward: 5′-GTTCTATGCGGTGGGCACT-3′, reversed: 5′-AACGAGGACCACTGAGGAAA-3′).

### Genotyping

SNP genotyping for *NRAP* variant was performed using TaqMan probes (Life Technologies) according to manufacturer protocol on QuantStudio 12 K Flex Real-Time PCR System (Applied Biosystems) in 231 unrelated DCM patients.

### Western blots

Heart tissue were sampled in ice-cold PBS, snap-frozen in liquid nitrogen and stored at −80 °C. Control sample was taken from heart transplantation recipient with available WES data and no truncating variants in *NRAP* gene. For Western blotting tissues were lysed with lysis buffer (50 mM HEPES pH 7.4, 1.0% Triton X-100, 150 mM NaCl, 10% glycerol, 5 mM EDTA) supplemented with Complete®protease inhibitors (Roche, Mannheim, Germany). Protein concentration was measured using Bio-Rad Protein Assay (Hercules, CA, USA). After SDS-PAGE proteins were transferred to Protran nitrocellulose membranes (Schleicher and Schuell BioScience, Dassel, Germany). Membranes were blocked and incubated with primary antibodies for: NRAP (Abcam, ab122427) and β-actin (Sigma-Aldrich, A3854) according to the manufacturers’ protocols. After extensive washing with TBST, the membranes were incubated for 40 min with HRP-linked secondary antibodies (Cell Signaling Technology). The chemiluminescence reaction was visualized with Stella 8300 Bio-imager (Raytest, Straubenhardt, Germany).

## References

[1] Luo G (1997). Complete cDNA sequence and tissue localization of N-RAP, a novel nebulin-related protein of striated muscle. Cell Motil Cytoskeleton.

[2] Carroll SL, Horowits R (2000). Myofibrillogenesis and formation of cell contacts mediate the localization of N-RAP in cultured chick cardiomyocytes. Cell Motil Cytoskeleton.

[3] Lu S, Borst DE, Horowits R (2008). Expression and alternative splicing of N-RAP during mouse skeletal muscle development. Cell Motil Cytoskeleton.

[4] Herrera AH, Elzey B, Law DJ, Horowits R (2000). Terminal regions of mouse nebulin: sequence analysis and complementary localization with N-RAP. Cell Motil Cytoskeleton.

[5] Mohiddin SA (2003). Genomic organization, alternative splicing, and expression of human and mouse N-RAP, a nebulin related lim protein of striated muscle. Cell Motil Cytoskeleton.

[6] Zhang JQ (2001). Ultrastructural and biochemical localization of N-RAP at the interface between myofibrils and intercalated disks in the mouse heart. Biochemistry.

[7] Luo G, Herrera AH, Horowits R (1999). Molecular interactions of N-RAP, a nebulin-related protein of striated muscle myotendon junctions and intercalated disks. Biochemistry.

[8] Lu S, Carroll SL, Herrera AH, Ozanne B, Horowits R (2003). New N-RAP-binding partners alpha-actinin, filamin and Krp1 detected by yeast two-hybrid screening: implications for myofibril assembly. J Cell Sci..

[9] Ehler E (2001). Alterations at the intercalated disk associated with the absence of muscle LIM protein. J Cell Biol..

[10] Lu S (2011). Cardiac-specific NRAP overexpression causes right ventricular dysfunction in mice. Exp Cell Res..

[11] Perriard JC, Hirschy A, Ehler E (2003). Dilated cardiomyopathy: a disease of the intercalated disc?. Trends Cardiovasc Med..

[12] Nagy E, Maquat LE (1998). A rule for termination-codon position within intron-containing genes: when nonsense affects RNA abundance. Trends Biochem Sci..

[13] Grzybowski J, Bilinska ZT, Janas J, Michalak E, Ruzyllo W (2002). Plasma concentrations of N-terminal atrial natriuretic peptide are raised in asymptomatic relatives of dilated cardiomyopathy patients with left ventricular enlargement. Heart.

[14] Bielecka-Dabrowa A (2016). Differences in biochemical and genetic biomarkers in patients with heart failure of various etiologies. Int J Cardiol..

[15] Bielecka-Dabrowa A. *et al*. The influence of atorvastatin on parameters of inflammation left ventricular function, hospitalizations and mortality in patients with dilated cardiomyopathy–5-year follow-up. *Lipids Health Dis*. Apr 8; **12** 47 (2013). 10.1186/1476-511X-12-47 PMC3641983 23566246

[16] Bielecka-Dabrowa A (2013). Heart failure biomarkers in patients with dilated cardiomyopathy. Int J Cardiol..

[17] D’Avila F (2016). Exome sequencing identifies variants in two genes encoding the LIM-proteins NRAP and FHL1 in an Italian patient with BAG3 myofibrillar myopathy. J Muscle Res Cell Motil..

[18] Ploski R (2014). Does p.Q247X in TRIM63 cause human hypertrophic cardiomyopathy?. Circ Res..

